# Growing implementation scientists: NHLBI’s K12 programs in heart, lung, blood, and sleep research

**DOI:** 10.1186/s43058-026-01027-5

**Published:** 2026-07-01

**Authors:** Shahnaz B. Khan, Cara C. Lewis, Padma T. Mudakala, Keith A. Mintzer, Jennifer S. Curry, George A. Mensah

**Affiliations:** Center for Translation Research and Implementation Science National Heart, Lung, and Blood Institute, National Institutes of Health, Bethesda, MD USA

**Keywords:** Implementation science, Training, Career development, K12, Scholars, Translational research

## Abstract

**Background:**

Implementation science seeks to accelerate the adoption, integration, and scale-up of evidence-based interventions (EBIs) to improve health and reduce disparities. As the field expanded, demand for trained implementation scientists rapidly outpaced workforce capacity. To address this need, the National Heart, Lung, and Blood Institute (NHLBI) launched the institutional Research Career Development Programs in T4 Implementation Research (K12) Next Generation of Implementation Science (iNexIS) in 2016. The program aimed to expand the national pool of rigorous implementation researchers prepared to conduct late-stage translation (T4) studies focused on heart, lung, blood, and sleep (HLBS) conditions. This report presents early outcomes from the inaugural K12 implementation science cohort to assess preliminary workforce growth and productivity.

**Methods:**

A retrospective cohort analysis was conducted using NIH RePORTER, Query/View/ Report (QVR), and Research Performance Progress Reports (RPPRs) for all awards funded through RFA-HL-17-016. Data extracted (through September 2025) included institutional characteristics, scholar demographics, training duration, grant applications and awards, and peer-reviewed publication output. Productivity measures encompassed: (1) Career Development Awards (CDAs), (2) Research Project Grants (RPGs) and equivalents, and (3) Loan Repayment Program (LRP) applications/awards. Descriptive statistics were used to characterize scholar output, time to first CDA or RPG, and cumulative citation metrics.

**Results:**

Ten institutions were funded, supporting 75 scholars across diverse disciplines, primarily pulmonary/critical care medicine (16%), internal medicine (15%), and pediatrics (13%). Most scholars were women (58%); 42% held PhDs and 32% held MDs. Of 215 grant submissions, 95 awards were made to 54 scholars (44% success rate overall; 36% excluding LRPs). Scholars achieved 32 RPGs (24% success rate across RPG submissions) and 27 CDAs. Median time to CDA was 31 months, and to RPG was 43 months—shorter than typical NIH-wide timelines. Scholars collectively generated an estimated 393 publications with a median Relative Citation Ratio of 1.64, reflecting strong research influence. Post-training positions span academic health centers, VA medical centers, community health organizations, research institutes, and industry settings across 17 states.

**Conclusions:**

Early findings suggest that NHLBI’s iNexIS K12 program effectively expanded the implementation science workforce, accelerated progression to independent funding, and fostered high research productivity. While long-term impact will require follow-up at beyond 10-years post-program, current metrics indicate strong return on investment. Continued support for structured implementation science training programs remains critical to meet growing workforce demands and advance late-stage translational research.

**Supplementary Information:**

The online version contains supplementary material available at https://doi.org/10.1186/s43058-026-01027-5.

## Contributions to the literature

- Reports outcomes from 75 scholars across 10 institutions, providing evidence on implementation science workforce development within a heart, lung, blood, and sleep (HLBS) focused K12 program.
- Establishes empirical benchmarks for implementation science career growth by scholars achieving a 44% overall grant success rate, median 31 months to career development awards (CDAs), and 43 months to research projects awards (RPGs).
- Documents accelerated transitions to independent funding, with 19 scholars moving directly from K12 support to research project grants.
- Reinforces the continued need to invest in implementation training programs to create pathways for preparing the next generation of translational researchers.

## Introduction

Implementation science emerged, in response to the well-documented gap between research and practice, as a field of inquiry into methods or strategies that accelerate the adoption and integration of evidence-based interventions (EBIs). Its purpose is to translate scientific discovery and intervention research into population health gains and reduces disparities. To drive research in this field, rigorous education and training opportunities are necessary to grow a capable workforce. To this end, in 2011, the NIH launched the competitive Training Institute for Dissemination and Implementation Research in Health (TIDIRH) [1], producing a cadre of implementation scientists across its 27 Institutes, Centers, and Offices (ICOs). Despite this, demand for implementation science expertise still outpaced workforce growth, and training needs for ICO mission areas remained.

In parallel, several NIH ICOs developed dedicated implementation science units to support the field’s growth. In 2014, the National Heart, Lung, and Blood Institute (NHLBI) launched the Center for Translation Research and Implementation Science (CTRIS) to stimulate late-stage translation research for heart, lung, blood, and sleep (HLBS) conditions [2]. Through a training portfolio analysis, CTRIS observed a need for mechanisms to grow implementation scientists capable of conducting HLBS-focused T4 translational research. In 2016, NHLBI launched a Request for Applications (RFA) [3] to fund institutional career development grants to develop implementation scientists through the Research Career Development Programs in T4 Implementation Research (K12) Physician Scientist Award Program.

The K12 program aimed to develop the workforce by engaging mentors from various sectors and multiple disciplines. This structured, team-based career development program fostered implementation science independence in investigators studying EBIs for HLBS conditions, with the ability to drive transdisciplinary research. Under the K12 mechanism, awards are made to institutions (rather than individuals) based on their training environments, with potential scholars applying to the institutions (rather than directly to NHLBI) for consideration. Thus, institutional award impact is reflected in the diverse range of investigators, including physician-scientists and non-clinician PhD researchers. This impact is typically measured by the proportion of scholars subsequently obtaining an individual career development award (CDA) or a research project grant (RPG)/RPG equivalent. Nearly a decade since its launch, this brief report presents preliminary findings from the NHLBI K12 implementation science training program, including scholar demographics, productivity, and short-term outcomes.

## Methods

### Overview NHLBI K12: building the next generation of implementation science (iNexIS)

Institutions typically funded two new scholars per year, each receiving up to 3 years of support for consecutive 12-month appointments, including salary, mentorship, research training, grant writing, and research expenses and education up to $100,000 annually. Participation required 75% protected time for implementation science. Scholars entered the program from July 1, 2018, with the last finishing on June 30, 2024. NHLBI hosted annual meetings in conjunction with the AcademyHealth/NIH Dissemination and Implementation Research in Health Conference in Washington DC in 2018 and 2019 with mentors, scholars, administrators, and federal team members to optimize approaches for mentoring, curriculum, technology, teaching, and methodologies. The 2020 meeting, held virtually due to the COVID-19 pandemic, continued this focus.

### Design, data collection, and analysis

We conducted a retrospective cohort study of institutions and scholars in the iNexIS K12 program using NIH RePORTER [4] and NIH’s internal Query/View/Report (QVR) system to identify awards made via RFA-HL-17-016. QVR reports provided grant submission and award history and publications records. Each K12’s Research Performance Progress Report (RPPR) was reviewed for scholar details including sex, race/ethnicity, degree, professional/clinical training, project focus, and publications, which were included as a measure of scholar productivity. Publication records were further supplemented by data retrieved from QVR. QVR reports provided grant submission and award history and publications records.

As of September 5, 2025, data were retrieved on institutional location, scholars trained per institution, demographics, grant submissions, funding success, and publications. Productivity was measured by Career Development Awards (CDAs) (e.g., IK2, K01, K08, K23), Research Project Grants (RPGs) or equivalents, (e.g., I01, I21, R01, R03, R18, R21, R34, R56, R61, U18, UG3), and Loan Repayment (LRP) (e.g., L30, L32, L40, L60) applications and awards. Grant-related information for NIH, AHRQ, and the VA were included only for grants on which the scholar served as Principal Investigator (PI). Grants on which they were a co-investigator or multiple-PI (MPI) were excluded. Descriptive statistics characterized productivity, time to first award, success rate, and publication count.

## Results

### K12 institutions and scholar demographics

**Fig. 1:**
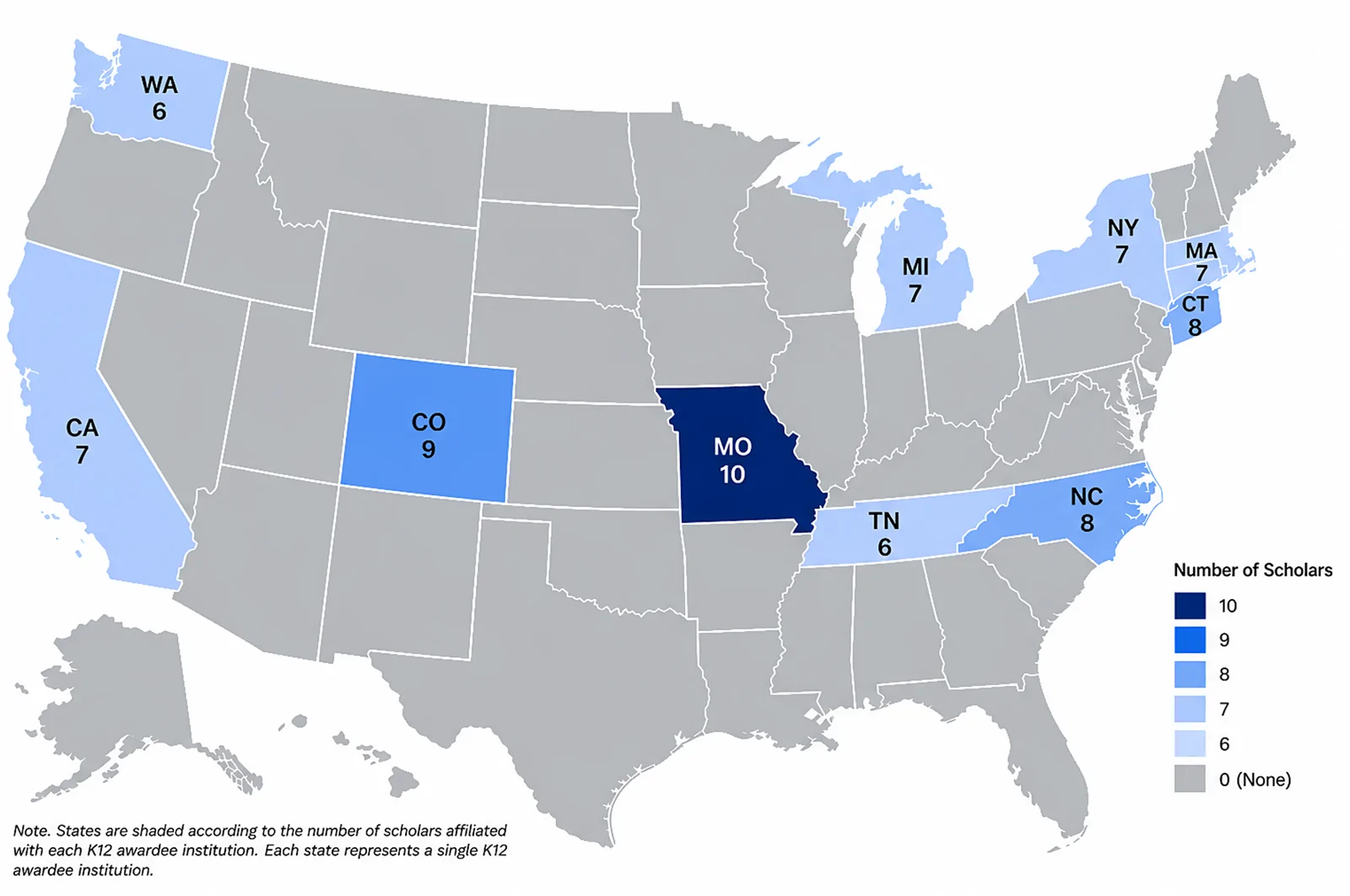
Geographic distribution of scholars across K12 awardee institutions (*N* = 75). States are shaded according to the number of scholars affiliated with each K12 awardee institution. Each state represents a single K12 awardee institution. Numbers indicate the number of scholars per state

Ten institutions received 5-year grants in 2017, totaling $2.84 million per institution in Direct Costs. Six completed the program in 2022; the remainder used no-cost extensions with the last finishing on July 30, 2024. Figure 1 shows the geographical distribution of scholars trained at K12 awardee institutions across the United States.

Seventy-five scholars participated across programs. Table 1 summarizes demographics, sex, degree, and professional/clinical discipline. Most scholars were female (58%), with the largest groups identifying as White non-Hispanic (42%) and Asian non-Hispanic (13%).

**Table 1:** Characteristics of scholars participating in the K12 program (*n* = 75)

Demographics upon entry to the K12 program	*n*	(%)
**Sex**		
Female	43	58
Male	13	17
Unreported	19	25
**Race/ethnicity**		
White and Non-Hispanic	31	42
Asian and Non-Hispanic	10	13
Hispanic	3	4
African-American/Black and Non-Hispanic	4	5
More than one Race and Hispanic	2	3
Unreported	25	33
**Professional/Clinical Discipline**		
Pulmonary & Critical Care Medicine	12	16
Internal Medicine	11	15
Pediatrics	10	13
Health and Behavioral Sciences	9	12
Population and Public Health Sciences	7	9
Health Services	5	7
Pharmacy	4	5
Nursing	4	5
Emergency Medicine	4	5
Nutrition	3	4
Internal Medicine & Pediatrics	3	4
Engineering	1	1
Health Policy & Health Economics	1	1
Occupational Therapy & Rehabilitation	1	1
**Post-graduate degree(s)**		
PhD	31	42
MD	24	32
MD + PhD	3	4
MD +Master’s	10	13
PhD + MPH	1	1
Other (MS, DO, DSC, MPH)	6	8

Demographic data were missing or unreported for a subset of scholars with 25% for sex and 33% or race/ethnicity which limits interpretation of the full representativeness of the cohort. Scholars represented disciplines including pulmonary and critical care medicine (16%), internal medicine (15%), and pediatrics (13%). Nearly half held PhDs (42%) and a third held MDs (32%). Scholars are now distributed across 17 states, reflecting a broad national footprint across a range of settings including academic health centers, VA medical centers, community health organizations, research institutes, and industry settings.

### Scholar output

**Fig. 2:**
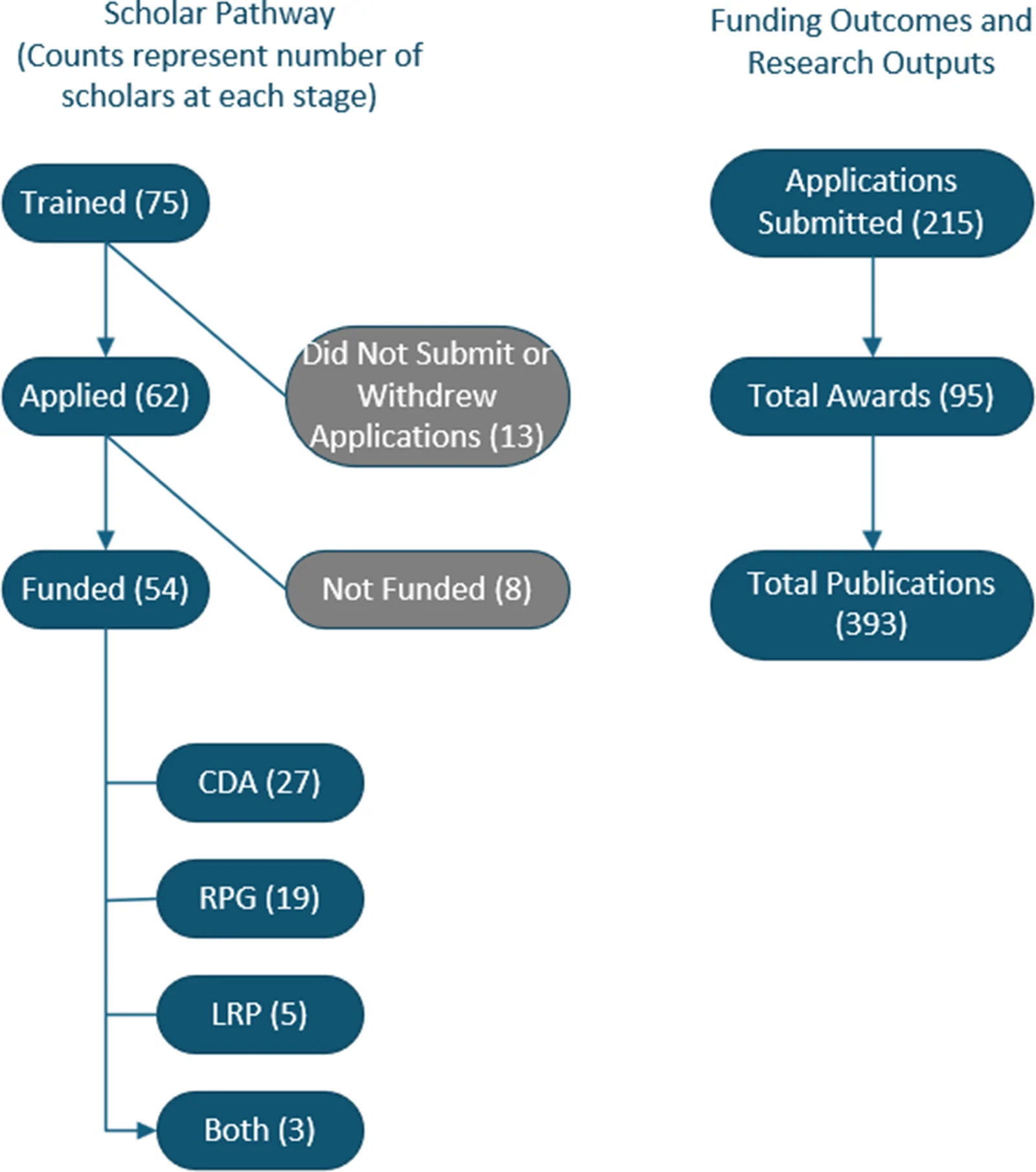
From scholar training to research impact. The left panel shows the number of scholars at each stage of the training and funding pathway, while the right panel summarizes resulting grant outcomes and research outputs. CDA, career development award; RPG, research project grant, LRP, Loan Repayment

Of the 75 scholars, 62 (83%) submitted a total of 215 grant applications across all mechanisms – Career Development Awards (CDAs), Research Project Grants (RPGs), and Loan Repayment Programs (LRPs), resulting in a 44% overall success rate (95 awards for 215 applications). Excluding LRPs, the success rate was 36% (62 awards from 171 applications). Figure 2 displays scholar output by applications, awards, and publications. Of the 75 scholars, 13 did not have any awarded grants to report within the study follow-up period.

**Fig. 3:**
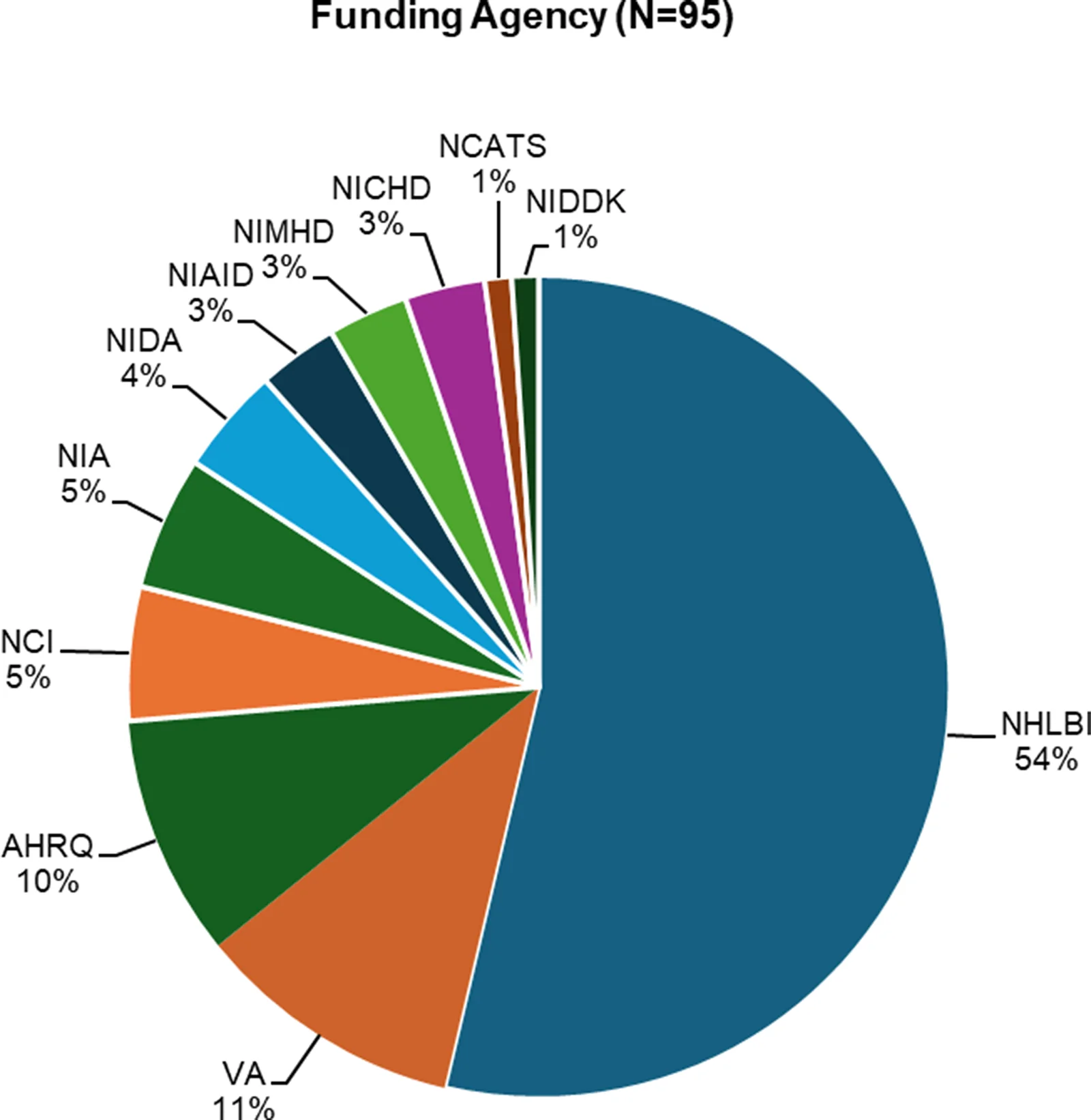
Funding Agency of Scholars’ Awards. Abbreviations: NHLBI, National Heart, Lung, and Blood Institute; VA, Veterans Affairs; AHRQ, Agency for Healthcare Research and Quality; NCI, National Cancer Institute; NIA, National Institute on Aging; NIDA, National Institute on Drug Abuse; NIAID, National Institute of Allergy and Infectious Diseases; NIMHD, National Institute on Minority Health and Health Disparities; NICHD, National Institute of Child Health and Human Development; NCATS, National Center for Advancing Translational Sciences; NIDDK, National Institute of Diabetes and Digestive and Kidney Diseases

**Fig. 4:**
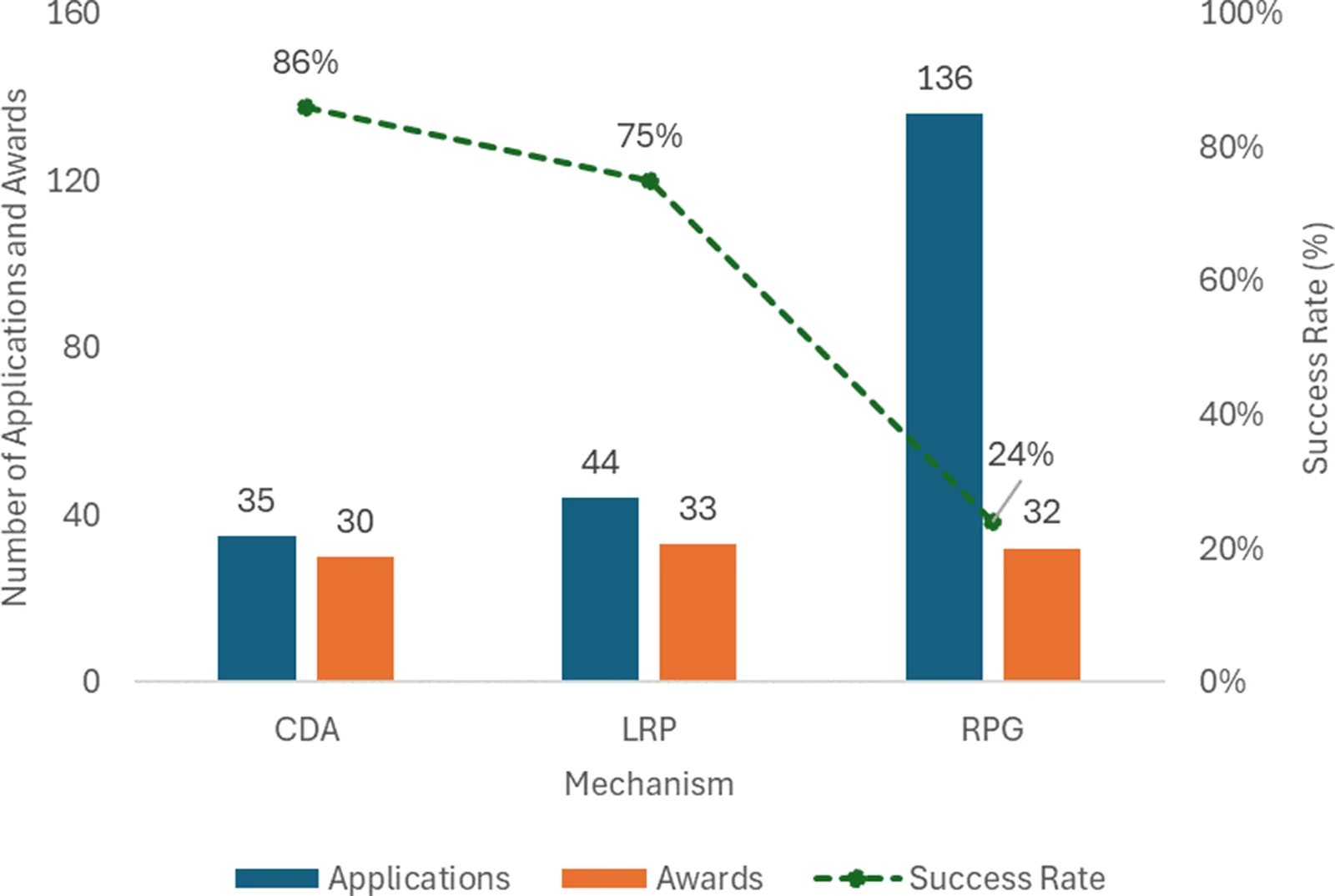
Success rate of scholars’ grant applications by mechanism. Number of scholar applications and awards by grant mechanism (CDA, LRP, RPG), with success rates (%) indicated for each category. Mechanisms include Career Development Awards (CDA: IK2, K01, K08, K23), Loan Repayment Program (LRP), and Research Project Grants (RPG: I01, I21, R01, R03, R18, R21, R34, R56, R61, U18, UG3)

80% of awards were NIH-funded, with 54% from NHLBI and 20% from VA and AHRQ (Fig. 3). Awards spanned three mechanisms: LRPs (35%), RPGs (34%), and CDAs (31%), with a range of activity codes within each category (Table 2**).** Of the 171 non-LRP submissions, 136 were RPG submissions, yielding 32 awards and 24% success rate (Fig. 4).

**Table 2:** Grant type and activity codes for awarded projects (***n* = 95)**

	*n*	(%)
*Research Project Grants (RPGs)/RPG Equivalent*	32	34
Type		
R01	8	9
R21	6	7
I01	5	5
R18	4	4
R03	2	2
R34	2	2
R61	1	1
R56	1	1
U18	1	1
UG3	1	1
I21	1	1
*Loan Repayment (LRP)*	*33*	*35*
Type		
L30	21	22
L40	9	10
L32	2	2
L60	1	1
*Career Development Awards (CDAs)*	*30*	*31*
Type		
K23	18	19
K08	4	4
K01	4	4
IK2	4	4

Grant activity averaged 3.5 submissions per active scholar across the 62 scholars who submitted at least one application. Approximately 27% of submissions were resubmissions, which demonstrated a notably higher success rate compared to new submissions (57% vs. 40%), underscoring the importance of iterative application strategies. Grant activity was also geographically concentrated, with approximately 66% of submissions originating from the institutions where scholars completed their K12 training, suggesting continued affiliation with and productivity within prior training environments.

Time to CDA or RPG ranged from 1 to 7 years. Median time to CDA was 31 months and to RPG 43 months. Three scholars achieved both awards, a CDA and an RPG, with a median of 51 months from program entry to RPG (Table 3). Since launch, scholars have published an estimated 393 articles, with a median Relative Citation Ratio (RCR) of 1.64 indicating above-average citation impact relative to other papers in the same field and a cumulative weighted RCR of 574.84 [5].

**Table 3:** Cumulative time to award by mechanism among K12 scholars (*n* = 49)

	CDA	RPG	Both (CDA and RPG)
Number of Scholars	27/49 (55%)	19/49 (39%)	3/49 (6%)
Median Time to Award (from K12 entry)	31 Months	43 Months	51 months
1 year	5 (19%)	5 (26%)	0 (0%)
2 years	16 (59%)	9 (47%)	0 (0%)
3 years	22 (81%)	11 (58%)	0 (0%)
4 years	26 (96%)	16 (84%)	2 (67%)
5 years	27 (100%)	18 (95%)	3 (100%)
6 years	27 (100%)	18 (95%)	3 (100%)
7 years	27 (100%)	19 (100%)	3 (100%)

Cumulative time to award among K12 scholars (*n* = 49) by funding mechanism. Values represent the cumulative number and percentage of scholars within each mechanism group (CDA, *n* = 27; RPG, *n* = 19; both CDA and RPG, *n* = 3) who obtained an award by each time point following K12 program entry. Median time to award is reported in months. CDA, career development award; RPG, research project grant

## Discussion

### Was the iNexIS K12 worth the investment?

The median time for investigators to obtain an NIH R01 is approximately 12 years [6]; success of NHLBI’s iNexIS would ideally accelerate time to independence. While return on investment and long-term impact will be best assessed in 2034 when the final cohort of scholars are at least 10 years beyond completion of the program, preliminary data suggest several positive indicators of success. Nineteen scholars (31%) moved directly from K12 to RPGs, suggestive of the program’s ability to drive an accelerated timeline to independence.

**Fig. 5:**
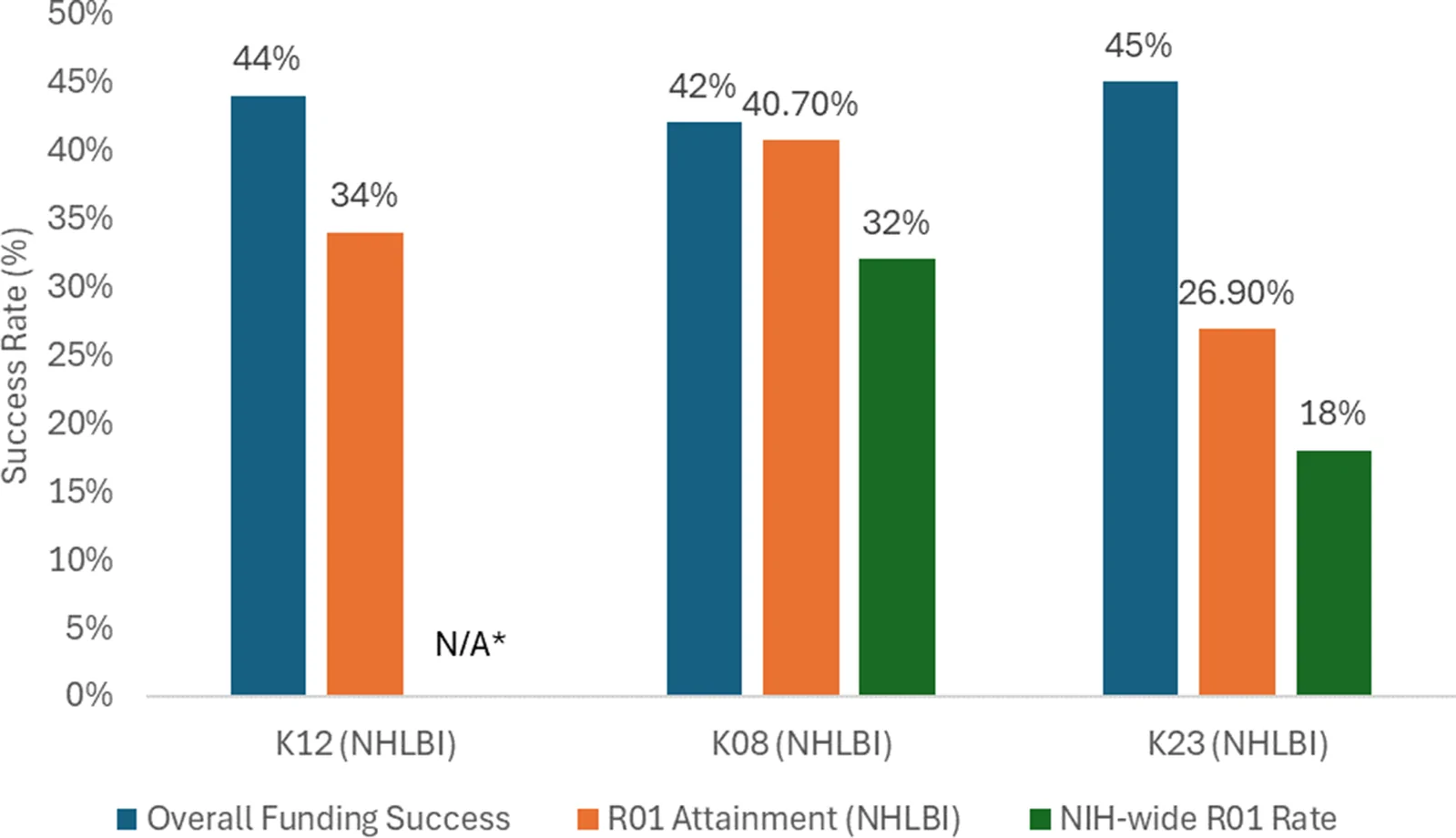
Overall funding success and R01 attainment among NHLBI K12, K08, and K23 awardees. *NIH-wide R01 rates not available for K12. Overall funding success (defined as receipt of any subsequent research grant) and R01 attainment rates are shown for K12 scholars and NHLBI K08 and K23 awardees. K12 scholars achieved an overall funding success rate of 44% and an R01 attainment rate of 34%. Among NHLBI K08 and K23 awardees, investigators demonstrated overall funding success rates of 42% and 45%, respectively, with corresponding R01 attainment rates of 40.7% and 26.9%. NIH-wide R01 success rates for K08 and K23 awardees (32% and 18%, respectively) are included for comparison. NIH-wide R01 attainment rates are not available for K12 and are therefore not shown

The iNexIS K12 demonstrated strong productivity relative to established benchmarks. First, the iNexIS K12 supported 1.7 times as many scholars (75 vs. 43) at 21% lower per-scholar cost than a prior NHLBI K12 in emergency care [7], based on total direct costs across each program’s award period. Next, the 44% overall award success rate (36% after excluding LRPs) for obtaining any grant funding after the K12 is comparable to previous NHLBI published analyses of K08 (42%) and K23 (45%) awardees [8]. For R01 success, iNexIS K12 scholars demonstrated a success rate of 34%, while NHLBI K08 and K23 awardees achieved 40.7% and 26.9%, respectively—both exceeding NIH-wide success rates for obtaining R01s among K08 (32%) and K23 (18%) awardees (Fig. 5).

Career progression among iNexIS scholars also reflected meaningful advancement toward independence. Twenty-seven of the 75 K12 scholars progressed to individual K awards, gaining an additional 3–5 years of protected time to engage in tailored training and emerge with robust pilot data for an RPG application. Time to award among iNexIS scholars was 31 months to CDA and 43 months to first RPG. These timelines compare favorably to benchmarks from the broader extramural community. A large NIH-wide analysis of 6,744 K01/K08/K23 awardees found a median time of 60 months to first RPG and 67 months to first R01 among awardees [9]. Among scholars in a prior NHLBI K12 program in emergency care research, the median time to first RPG was 50 months from program entry [8], compared to 43 months among iNexIS scholars. At the National Cancer Institute (NCI), the median time from PhD degree to first R01 doubled to approximately 12 years in recent cohorts [10], further underscoring the accelerated trajectory observed among iNexIS scholars.

Importantly, NHLBI invested in this K12 to not only increase the number of implementation scientists driving cutting-edge research accelerating integration of HLBS EBIs, but also to grow the breadth of expertise and reach of new experts in late-stage translation. The research topics of Scholars’ subsequent CDA/RPG awards include genomic medicine, artificial intelligence, health impacts of extreme weather, non-communicable disease disparities, digital health for chronic lung disease, and system level critical care operations. These examples highlight the breadth of methodological and substantive expertise cultivated by iNexIS, extending beyond traditional disciplinary boundaries. Some scholars have emerged as national and international leaders in the field, as evidenced by appointments to roles such as chief of infectious diseases at major academic medical centers and VA healthcare systems, director of institutional implementation science training programs, associate program director of clinical research fellowships, and lead investigator on multi-site national trials. Several scholars have also taken on advisory roles with federal agencies including the NIH and the WHO and have extended their implementation science expertise into emerging areas such as artificial intelligence (AI) and clinical decision support. Scholars reach now extends beyond the initial 10 awardee institutions, spreading to 17 states across the United States spanning a mix of academic health centers, VA medical centers, community health organizations, research institutes, and industry settings.

### Training and career development mechanisms for implementation science

Training and career development in implementation science are vital for accelerating public health impact across HLBS conditions. Training implementation scientists capable of rigorous, replicable, and cutting-edge research is key to translating EBIs for populations in need. However, since this one-time RFA, CTRIS and NHLBI have relied on parent PARs for supporting training and career development in implementation science. In a 10-year (2013–2023) portfolio analysis of NHLBI supported implementation science research, 236 D&I focused K applications (K01, K08, K12, K23, K24, K99) were submitted, of which 123 were funded.

A comprehensive list of funding opportunities available to the extramural community as of November 25, 2025, designed to support training and career development (e.g., K08, K23, K24) is provided in Additional File 1: Table S1. These invite investigator-initiated research in which a broad array of topics can be proposed, with implementation science called out explicitly in NHLBI’s sixth Strategic Objective (Optimize clinical and implementation research to improve health and reduce disease). Other mechanisms, such as the R36 used by AHRQ for predoctoral dissertation or the T35 could be creatively leveraged for implementation science training. The NHLBI uses the T35 funding opportunity to support the summer research training of health professional students for whom a program focused on implementation science for HLBS could be quite valuable. Another mechanism that may be underutilized by NHLBI-funded investigators is the R25, which the NIMH, with support from NIDA and the Department of Veterans Affairs (VA), successfully used to fund “The Implementation Research Institute (IRI)” at Washington University in St. Louis [11]. This highly successful competitive, cohort-based model trained over 100 fellows across 15 years [12]. It is important to note that the R25 eligibility criteria and permissible program features vary across NIH ICOs; investigators interested in pursuing this mechanism should consult current NOFOs and engage directly with NHLBI program officers to determine whether the R25 can support the specific features of their proposed training program. Washington University launched a complementary program focused on HIV, Infectious Disease, and Global Health Implementation Research with funding from NIH’s Fogarty International Center and Viiv Healthcare in 2021. CTRIS is eager to provide technical assistance to investigators interested in pursuing funding opportunities to train implementation scientists for HLBS. Indeed, NHLBI remains committed to programs that train future biomedical researchers, as underscored by NIH’s Director Statement on Advancing NIH’s Mission Through a Unified Strategy [13].

### Environmental scan of D&I training opportunities

To contextualize the NHLBI iNexIS K12 program within the broader training landscape, we conducted a preliminary environmental scan of D&I training opportunities developed by other organizations outside of NIH since 2015. Programs were identified through searches of organizational websites, conference proceedings, and the published literature, focusing on initiatives offering structured training in dissemination and implementation science. This review highlighted a diverse set of training and capacity-building initiatives across universities, professional societies, research networks, and international groups, ranging from short-format workshops to longer mentored training experiences. Several of these programs remain active and continue to strengthen the field’s workforce. For instance, Washington University, the University of Pennsylvania, and Northwestern University all offer 1–3-day non-competitive training institutes. Adult & Child Center for Outcomes Research & Delivery Science (ACCORDS) at the University of Colorado Anschutz regularly hosts workshops and short courses, as does Dartmouth’s Center for Implementation Science. The Veterans Health Administration (VHA) Quality Enhancement Research Initiative (QUERI) Implementation Science Training Program also represents a sustained investment in preparing clinicians and researchers to apply D&I methods in real-world healthcare settings. While these initiatives demonstrate durable institutional commitment, the majority are short-format and non-mentored, limiting their ability to support scholars through the full arc of career development—from early training to independent funding.

These findings align with recent global scans of D&I training opportunities, which identified a fragmented training landscape with growing demand but insufficient capacity to meet workforce needs [14, 15]. Our scan reinforces these conclusions in the HLBS context specifically, revealing persistent gaps, including limited access to long-term, mentored career development pathways and uneven training opportunities across regions and disciplines. What distinguishes structured, mentored programs like the iNexIS K12 is their ability to provide protected time, sustained mentorship, and institutional infrastructure—conditions that short-format programs cannot replicate and that are likely necessary to produce investigators capable of leading independent research programs. Sustaining such programs requires dedicated funding mechanisms and institutional commitment; the shift to parent training Program Announcements (PAs) following iNexIS RFA underscores the vulnerability of this model without continued targeted investment. Collectively, these observations reinforce the case for sustained, structured implementation science training programs to expand the number of trained investigators and the breadth of the expertise needed to advance late-stage translational research for HLBS conditions.

This ongoing need for implementation science training programs exists in the context of a changing federal funding landscape. NIH is currently undergoing Notice of Funding Opportunity (NOFO) consolidation to streamline and clarify funding opportunities for the extramural community and to simplify the requirements for peer review now that it is centralized under the Center for Scientific Review (CSR) at NIH Office of the Director. To complement this, NIH has launched a Clearinghouse for Highlighted Topics (HTs) where ICOs, can express interests that are priorities for investigator-initiated research annually. Prospective applicants should regularly consult the Clearinghouse and www.grants.gov [16] for updated training and career development funding opportunities that can support implementation science.

Future evaluations of implementation science capacity building programs would benefit from a broader set of metrics beyond the administrative productivity measures reported here. These include qualitative assessment of scholar confidence and competency in implementation science methods; tracking of D&I focused publications and grants as a proportion of total scholarly output; measures of mentorship activity, including whether iNexIS scholars have gone on to mentor the next generation of implementation scientists; community and policy engagement outcomes; and measures of the extent to which scholars’ research addresses health disparities in underserved populations. Longitudinal surveys of scholars and their mentors, as employed in prior K12 evaluations such as Morris et al. [8], could provide valuable complementary data to the administrative records used and offer a more complete picture of program impact over time.

There are several limitations to acknowledge. As the only published outcomes report from an NIH-funded implementation science K12 program, benchmarks for direct comparisons are unavailable, limiting our ability to determine whether findings are representative of implementation science career development programs broadly. Second, this secondary data analysis relied on administrative data sources (RPPRs, RePORTER, and QVR), which may introduce incomplete capture of scholar productivity; however, QVR data were cross-referenced against RPPR records to improve completeness and consistency. RPPRs are submitted directly by institutions to NHLBI and undergo federal review, providing a degree of institutional accountability. Systematic independent validation was not feasible given the administrative nature of the data and we were not able to conduct any follow-up with institutions directly. Third, grant productivity was assessed only for awards on which scholars served as principal investigator, excluding multi-PI grants, which may undercount overall scholarly output. Fourth, we were unable to systematically classify publications and RPGs by implementation science focus, precluding assessment of the proportion of scholarly output directly aligned with the program’s core mission; this remains an important area for future evaluation. Fifth, the follow-up period for many scholars remains relatively short and return on investment and long-term impact will be more meaningfully assessed in 2034, when the final cohort of scholars are at least 10 years beyond program completion.

## Conclusions

Early signals from NHLBI’s iNexIS K12 program suggest meaningful short-term impact in building the implementation science workforce. CTRIS will reassess the longer-term impact once scholars have had sufficient time to complete their training, generate new applications, and make their way through the NIH review and award cycle. Although NHLBI did not renew this particular K12 program for another round, CTRIS remains committed to growing the number of implementation scientists and related niche areas of expertise including economic evaluation, systems science, data science & informatics, and community-engaged research. The demand for implementation science is likely to continue to outpace the capacity of the current workforce as NIH seeks to evaluate public health impact more rigorously with future investments [17].

## Electronic supplementary material

Below is the link to the electronic supplementary material.

Supplementary Material 1

## Data Availability

The datasets generated and/or analyzed during the current study are available in the [NIH RePORTER] (https://www.reporter.nih.gov). Some data analyzed during the current study are not publicly available in order to protect the privacy of scholars.

## Abbreviations

ACCORDS: Adult & Child Center for Outcomes Research & Delivery Science
AHRQ: Agency for Healthcare Research and Quality
CDA: Career Development Award
CSR: Center for Scientific Review
CTRIS: Center for Translation Research and Implementation Science
D&I: Dissemination and Implementation
EBI: Evidence-Based Intervention
HLBS: Heart, Lung, Blood, and Sleep
HT: Health Topic
ICO: Institute, Center, and Office
iNexIS: Next Generation of Implementation Science
LRP: Loan Repayment Program
MPI: Multiple Principal Investigator
NCI: National Cancer Institute
NHLBI: National Heart, Lung, Blood, and Sleep Institute
NIDA: National Institute on Drug Abuse
NIH: National Institutes of Health NOFO: Notice of Funding Opportunity
PA: Program Announcement
PI: Principal Investigator
QVR: Query/View/Report
QUERI: Quality Enhancement Research Initiative
RCR: Relative Citation Ratio
RFA: Request for Applications
RPG: Research Project Grant
RPPR: Research Performance Progress Report
T4: Late-Stage Translation Research
TIDIRH: Training Institute for Dissemination and Implementation Research in Health
VA: Veterans Affairs
VHA: Veterans Health Administration
WHO: World Health Organization
