# Supplementary material for: Growing implementation scientists: NHLBI’s K12 programs in heart, lung, blood, and sleep research
Source: Implement Sci Commun. 2026 Jul 1;7:192. doi: 10.1186/s43058-026-01027-5 (PMC13595544; doi:10.1186/s43058-026-01027-5)
Supplement: Supplementary file 1 [file 43058_2026_1027_MOESM1_ESM.docx]

**Additional Files**

**Additional File 1: Table S1. Active NHLBI Training and Career Development Opportunities for Implementation Science.**

| **Activity code** | **Title** | **Awardee** | **Trains** | **NOFO** |
| --- | --- | --- | --- | --- |
| K23 | Mentored patient-oriented research career development award | Individual | junior faculty members engaged in patient-oriented research | PA-24-185 (clinical trial not allowed and PA-24-184 (clinical trial required) |
| K24 | Midcareer investigator award in patient-oriented research | Individual | Associate professor or full professor within 10 years of their appointment as an associate professor | PA-24-187 (parent K24 independent clinical trial required) and PA-24-188 (parent K24 independent clinical trial not allowed) |
| K99/R00 | Pathway to independence award | Individual | Postdoctoral trainees transitioning into faculty positions | PA-24-194 (clinical trial not allowed)  PA-24-193 (independent clinical trial required) |
| K22 | Career transition award for intramural postdoctoral fellows and research trainees | Individual | NHLBI intramural postdoctoral trainees transitioning into faculty positions | PA-24-209 |
| K08 | Mentored clinical scientist research career development award | Individual | Junior faculty members | PA-24-181  (independent clinical trial required) PA-24-182 (clinical trial not allowed) |
| R03 | Small grant program for NHLBI K01/K08/K23 recipient clinical trial optional | Individual | NHLBI K01, K08, and K23 awardees | RFA-HL-25-011 |
| R25 | Short Courses on Innovative Methodologies and Approaches in the Behavioral and Social Sciences | Institutional | Graduate students, medical students, medical residents, postdoctoral fellows, early-stage investigators) established investigators (e.g., mid- and late-career researchers | RFA-OD-25-003 |
| T32 | Ruth L. Kirschstein national research service award | Institutional | Predoctoral and postdoctoral trainees | PA-25-168 |
| T35 | Ruth L. Kirschstein national research service award short term-institutional research training grant | Institutional | Students in health professional | PA-25-280 |
| F30 | Ruth L. Kirschstein individual predoctoral national research service award for MD/PHD and other dual degree fellowships | Individual | Students in dual-degree doctoral programs | PA-25-426 |
| F31 | Ruth L. Kirschstein predoctoral individual national research service award | Individual | Students in research doctoral programs. | PA-25-422 |
| F32 | Ruth L. Kirschstein postdoctoral individual national research service award | Individual | Doctoral degree recipients | PA-25-423 |

NOFO = Notice of Funding Opportunity. These NOFOs were available as of November 25, 2025.
